# Phloretin Protects Goat Adipose-Derived Mesenchymal Stem Cells Against Ferroptosis by Regulating the Nrf2/HO-1/GPX4 Signaling Pathway

**DOI:** 10.3390/ani16091286

**Published:** 2026-04-22

**Authors:** Yunan He, Minjuan Li, Zhongfa Wang, Chuanying Pan, Xianyong Lan, Weijun Guan

**Affiliations:** 1 Department of Animal Genetic Resources, Institute of Animal Science, Chinese Academy of Agricultural Sciences, Beijing 100193, China; henan99929@163.com (Y.H.); 17806280540@163.com (M.L.); 19722725957@163.com (Z.W.); 2 College of Animal Science and Technology, Northwest A&F University, Yangling 712100, China; panyu1980@126.com

**Keywords:** phloretin, AD-MSCs, ferroptosis, Nrf2/HO-1/GPX4 antioxidant pathway, ruminant breeding, lipid peroxidation

## Abstract

Ferroptosis of mesenchymal stem cells (MSCs) is a critical bottleneck restricting the efficiency of ruminant biological breeding. Phloretin, a natural bioactive polyphenol, exhibits potential ferroptosis-inhibitory activity. However, the regulatory effects and underlying mechanisms of phloretin on ruminant MSCs remain poorly understood. This study aimed to investigate the effects of phloretin on ferroptosis and elucidate its underlying molecular mechanisms. Herein, we isolated and cultured adipose-derived mesenchymal stem cells (AD-MSCs) from adipose tissue of a 9-day-old Leizhou goat and established a ferroptosis model in these cells using RSL3. We detected cell viability, proliferation, migration, ferroptosis-related indexes and key protein expression. The results showed that phloretin (25 and 50 μM) dose-dependently inhibited ferroptosis in goat AD-MSCs, reducing intracellular ferrous ion (Fe^2+^), reactive oxygen species (ROS) and lipid peroxidation levels, restoring glutathione content, and ameliorating mitochondrial structural damage. Mechanistically, phloretin exerted its anti-ferroptosis effects through direct antioxidant activity, activation of the Nrf2/HO-1/GPX4 signaling pathway and Fe^2+^ chelation. Nrf2 and GPX4 were key targets in this process. These results provide preliminary in vitro evidence and a theoretical basis for the potential application of phloretin in future research related to meat goat production and ruminant breeding.

## Simple Summary

This study investigated the protective effect of phloretin against ferroptosis in adipose-derived mesenchymal stem cells (AD-MSCs) from a Leizhou goat. Phloretin effectively alleviated RSL3-induced cell injury, reduced oxidative stress and lipid peroxidation, and enhanced cell proliferation and migration. These findings provide basic experimental evidence for the application of phloretin as a natural protective agent. This study also offers a new perspective for exploring effective strategies to improve stress resistance and health in ruminant breeding.

## 1. Introduction

Ruminants constitute the core taxon of global livestock production and a pivotal component of the agricultural economy, where key production traits including meat yield, reproductive efficiency, meat quality and stress resilience directly dictate breeding profitability and industrial competitiveness [1]. Traditional breeding approaches, mainly relying on phenotypic evaluation and genomic selection, have promoted genetic improvement in ruminants; however, they remain limited in the precise manipulation of complex biological processes such as cellular homeostasis [2]. In recent years, molecular breeding technologies—including somatic cell nuclear transfer, transgenic breeding, and stem cell-mediated tissue regeneration—have emerged as powerful tools to shorten breeding cycles and accelerate genetic gain [3]. Mesenchymal stem cells (MSCs) are a population of multipotent stem cells endowed with robust self-renewal, multilineage differentiation, and immunomodulatory and tissue regenerative capacities [4], and have been identified as essential cellular materials for ruminant breeding [5]. Elucidating the key molecular pathways that regulate MSC fate and translating them into viable breeding markers or nutritional intervention targets is therefore a critical research direction for advancing ruminant breeding technologies.

In ruminants (e.g., goats, sheep, and cattle), MSCs are intricately involved in embryonic development [5]. In vitro-expanded MSCs can serve as nuclear donor cells and seed cells, facilitating somatic cell nuclear transfer, transgenic cell screening, and other key breeding procedures. Studies in bovine somatic cell nuclear transfer have confirmed that MSCs significantly improve oocyte maturation and embryonic developmental efficiency [6]. Endogenous MSCs in vivo also modulate animal production performance by regulating muscle growth, fat deposition, reproductive organ development, and tissue repair [7]. Nevertheless, the proliferative activity and differentiation potential of MSCs are vulnerable to impairment during in vitro culture, in vivo transplantation, and exposure to environmental stressors. In vitro culture experiments using bovine MSCs have demonstrated that stressful conditions directly suppress their biological functions [8], which severely limits their application in genetic breeding and the improvement of production traits. Therefore, maintaining the viability, genomic stability, and functional integrity of both exogenous and endogenous MSCs is crucial for improving superior production traits in ruminants and advancing the sustainability of the livestock industry.

Nonetheless, MSC viability, proliferative activity and differentiation potential are markedly impaired in ruminants under in vitro culture expansion, in vivo transplantation and environmental stress; for example, in vitro co-culture experiments using bovine MSCs have demonstrated that stressful environments directly suppress their biological functions [8]. This severely limits their application in genetic breeding and the improvement of production traits.

Ferroptosis is a unique form of regulated cell death characterized by iron-dependent excessive accumulation of lipid peroxides. Accumulating evidence from stem cell transplantation studies indicates that the core molecular mechanism involves the collapse of the intracellular antioxidant defense system, most prominently the glutathione–glutathione peroxidase 4 (GSH-GPX4) axis [9]. As a major intracellular antioxidant, GSH functions synergistically with GPX4 to maintain redox balance, as established in classical mechanistic studies [10], and inactivation of this pathway is a central trigger of ferroptosis [9]. Ferroptosis has been recognized as a key driver of stem cell dysfunction. In vivo experiments in mice have revealed that ferroptosis directly impairs the biological effects of MSCs in vivo [11], and exerts profound effects on stem cell survival, stemness maintenance and differentiation fate by modulating oxidative stress, energy metabolism and cellular homeostasis [10,12]. Within the livestock breeding system, the ferroptosis susceptibility of MSCs is directly correlated with nuclear donor cell quality, cloned embryo developmental competence, screening efficiency of transgenic-positive cells, and individual developmental potential [9]. Moreover, the oxidative stress environment typical of intensive farming readily induces ferroptosis in endogenous MSCs, as verified by in vitro oxidative stress assays in bovine adipose-derived mesenchymal stem cells (AD-MSCs) [13], thereby weakening their regulatory roles in production traits. Consequently, ferroptosis in MSCs has become a major bottleneck restricting ruminant breeding efficiency. The identification of safe and effective ferroptosis inhibitors to improve the in vivo transplantation efficiency of MSCs through in vitro preconditioning is therefore of great practical significance for enhancing ruminant breeding efficiency.

Natural bioactive compounds have emerged as ideal interventional agents for stem cell protection and functional optimization owing to their high biocompatibility, clear molecular targets and ability to modulate cellular stress and death signaling cascades [14]. Phloretin, a natural dihydrochalcone polyphenol abundantly present in Rosaceae plants (e.g., apples and pears), exhibits diverse biological activities, such as potent antioxidation, reactive oxygen species (ROS) scavenging, iron chelation, lipid metabolism regulation, and anti-inflammation effects [15,16]. Preclinical studies have demonstrated that phloretin exerts robust ferroptosis-inhibitory and cytoprotective effects in various cellular models by activating the nuclear factor erythroid 2-related factor 2/heme oxygenase-1 (Nrf2/HO-1) antioxidant pathway, upregulating GPX4 expression and sustaining intracellular redox homeostasis [17]. Given its multiple biological activities, phloretin is a promising preconditioning agent to protect in vitro MSCs from ferroptotic damage. However, the regulatory effects, key molecular targets and underlying signaling mechanisms of phloretin on ferroptosis in ruminant MSCs remain largely unexplored.

From the perspective of the intrinsic correlation between genetic breeding and production traits, elite economic traits of ruminants (e.g., growth rate, carcass characteristics, meat quality, feed conversion efficiency and stress resistance) rely on the normal proliferation and differentiation of stem cells, as well as the subsequent development of tissues and organs [18,19]. Molecular studies on bovine intramuscular fat deposition have verified that AD-MSCs play a direct and pivotal role in regulating muscle growth, fat deposition, and energy metabolism in ruminants, and their functional integrity is a core determinant of individual growth and developmental potential [20]. In contrast, ferroptosis negatively regulates production traits by impairing stem cell activity, disturbing tissue repair, and destroying metabolic homeostasis [21]. Therefore, elucidating the regulatory mechanism of phloretin in MSC ferroptosis and developing potential pre-transplantation conditioning agents for MSCs hold important theoretical significance and practical application value for ruminant molecular breeding.

Building on the well-documented antioxidant and ferroptosis-inhibiting properties of phloretin from existing studies, we hypothesized that it may alleviate ferroptotic injury in MSCs by activating the Nrf2/HO-1/GPX4 antioxidant signaling pathway and suppressing the abnormal accumulation of iron-dependent lipid peroxidation.

As an economically important ruminant species, goats are widely regarded as an ideal animal model for exploring cellular biological processes and stress regulation mechanisms in ruminants. AD-MSCs, an important subtype of MSCs, have become the preferred cell model in ruminant stem cell research due to their easy isolation and minimal invasiveness [4]. Maintaining their functional integrity is crucial for improving economically important production traits in ruminants [21]. RSL3 is a specific inhibitor of GPX4. It covalently binds to the active site of GPX4, causing irreversible inactivation of GPX4 enzyme activity, and blocking the clearance of intracellular lipid peroxides. This leads to excessive lipid peroxide accumulation in cells, ultimately triggering ferroptosis [22]. In this study, an in vitro ferroptosis model of goat AD-MSCs was constructed using RSL3. We aimed to investigate the effects of phloretin on ferroptosis in AD-MSCs and its underlying molecular mechanisms, and to evaluate the potential value of phloretin as a pre-transplantation preconditioning agent for MSCs in ruminant genetic breeding and production performance improvement. The findings of this study are expected to provide novel molecular targets and theoretical references for the genetic breeding and healthy breeding of goats and other ruminants, establish a scientific basis for optimizing stem cell culture systems and developing efficient and safe pre-transplantation preconditioning reagents for MSCs, and ultimately promote animal reproductive performance and production efficiency.

## 2. Materials and Methods

### 2.1. Materials

Dulbecco’s Modified Eagle Medium/Nutrient mixture F-12 (DMEM/F12) basal medium, trypsin, Phosphate-Buffered Saline (PBS), and Fetal Bovine Serum (FBS) (Wuhan Sevier Biotechnology Co., Ltd., (Wuhan, China)). Penicillin–Streptomycin Solution (Double antibiotics) (Beijing Boshang Biotechnology Co., Ltd. (Beijing, China)). Antibodies against CD34, CD44, CD73, CD90, and CD105 (Beijing Soleibao Technology Co., Ltd., (Beijing, China)). Giemsa staining solution, Oil Red O staining kit, Alcian Blue staining kit (pH 2.5), and Alizarin Red S staining solution (Beijing Soleibao Technology Co., Ltd., (Beijing, China)). Phloretin (purity ≥ 98%) and RSL3 (purity ≥ 98%) (Beijing Zhongnong Sichen Biotechnology Co., Ltd., (Beijing, China)). The ferroptosis inhibitor Ferrostatin-1 (Sigma-Aldrich (Shanghai) Trading Co., Ltd., (Shanghai, China)). RIPA lysis buffer (Wuhan Sevier Biotechnology Co., Ltd., (Wuhan, China)) and BCA protein concentration determination kit (Beijing Soleibao Technology Co., Ltd., (Beijing, China)).

All animal operations were approved by the Animal Welfare and Ethics Committee of the Institute of Animal Science, Chinese Academy of Agricultural Sciences (Ethics Approval No.: IAS2025-222). All the AD-MSCs used in this study were isolated from the abdominal adipose tissue of one 9-day-old male Leizhou goat. No cell pooling from different donors was performed, and all subsequent experiments were conducted using AD-MSCs from this single donor with three technical replicates for each assay and three independent experimental repetitions.

### 2.2. Isolation and Identification of Cells

#### 2.2.1. Cell Isolation and Culture

Abdominal adipose tissue was collected from a 9-day-old male Leizhou goat and inoculated for cell culture. When the cell density reached 80–90% confluence, the cells were subcultured. AD-MSCs at passages 3 to 6 (P3-P6) were selected for all subsequent experiments. All AD-MSCs were cultured in a standard cell incubator at 37 °C with 5% CO_2_.

#### 2.2.2. Cell Identification

Immunofluorescence was used to identify the surface marker proteins of the goat AD-MSCs [8]. P4 AD-MSCs were seeded in confocal dishes, and when the cells reached 70% confluency, they were treated with corresponding primary and secondary antibodies, and the nuclei were stained with 4′,6-diamidino-2-phenylindole (DAPI). Finally, the cells were visualized using a confocal microscope (Leica Microsystems, Wetzlar, Germany). Reverse-transcription PCR (RT-PCR) was used to detect the expression levels of cell surface markers.

Oil Red O staining kit, Alcian Blue staining kit (pH 2.5), and Alizarin Red S staining solution were used to detect the multi-differentiation potential of the cells. The cells were cultured until the density reached approximately 80%, and then osteogenic induction medium (basal medium + FBS + 0.1 mg/mL double antibiotics + 0.6 μM dexamethasone + 10 mM β-glycerophosphate), adipogenic induction medium (basal medium + FBS + 0.1 mg/mL double antibiotics + 1.0 mM IBMX + 10 mg·L^−1^ insulin + 1.0 μM dexamethasone + 150 μM indomethacin), and chondrogenic induction medium (basal medium + FBS + 0.1 mg/mL double antibiotics + 0.2 μM dexamethasone + 40 μg·mL^−1^ ascorbic acid + 1% (1:100) ITS + 10 ng.mL^−1^ TGF-β3 + 10 μg.mL^−1^ IGF-1 + 0.5 mM sodium pyruvate + 50 μg/mL L-Proline) were added for directed induction differentiation. The medium was changed every 3 days until obvious characteristic changes appeared, and the corresponding staining solutions were added for observation.

### 2.3. Cell Viability Assay and Morphological Examination

Cell viability was measured using the CCK-8 assay (Shanghai Biyuntian Biotechnology Co., Ltd., (Shanghai, China)) to determine the cytoprotective effect of phloretin on goat AD-MSCs. All cell culture and incubation steps for the CCK-8 assay were performed under standard conditions of 37 °C and 5% CO_2_ in a humidified incubator. The cells were plated in 96-well plates and cultured until they reached approximately 80% density. Cells were treated with different concentrations of RSL3 (0.2–1.0 μM) and phloretin (10–150 μM) for 24 h. Cells treated with 0.1% served as controls. After incubation, 100 μL of CCK-8 working solution (10 μL CCK-8 stock solution + 90 μL basal medium) was added to each well in a CO_2_ incubator for 2 h. Finally, cell viability was evaluated by measuring the absorbance at 450 nm using a microplate reader. Based on the results, appropriate concentrations of RSL3 and phloretin were selected for subsequent experiments.

To detect the effect of phloretin on RSL3-induced goat AD-MSCs, the cells were seeded in 96-well plates and cultured until they reached approximately 80% confluence. After pretreatment with 25 μM and 50 μM phloretin for 4 h, RSL3 (0.6 μM) was added for further treatment for 24 h. CCK-8 working solution was added to each well and incubated in a CO_2_ incubator for 2 h. Finally, cell viability was evaluated by measuring the absorbance at 450 nm using a microplate reader. Morphological changes in AD-MSCs were observed and photographed under a microscope (Olympus, Tokyo, Japan).

### 2.4. Detection of ROS, Lipid Peroxidation, and Fe^2+^ Levels

Reactive oxygen species (ROS) detection kit and lipid peroxidation detection reagent (Shanghai Biyuntian Biotechnology Co., Ltd., (Shanghai, China)) and FerroOrange fluorescent probe (Nanjing Jiaozi Teng Scientific Equipment Co., Ltd., (Nanjing, China): According to the manufacturer’s instructions, the DCFH-DA fluorescent probe was used to detect ROS levels. DCFH-DA is a probe that can freely cross the cell membrane to detect intracellular ROS. The BODIPY-C11 probe was used to detect lipid peroxidation. The FerroOrange probe was used to detect Fe^2+^. After the corresponding treatment operations were performed according to the product instructions, observation and detection were carried out using a fluorescence microscope (Leica Microsystems, Wetzlar, Germany, Model: Leica DMi8) and a fluorescence microplate reader (Tecan Australia pty Ltd., Melbourne, Australia, Model: Infinite M200 pro). AD-MSCs were seeded in black-walled clear-bottom 96-well plates (Beijing Zhongyuan Taihe Biotechnology Co., Ltd., (Beijing, China)) for all fluorescence-based assays.

### 2.5. Measurement of GSH and MDA Levels

The malondialdehyde (MDA) detection kit and GSH and GSSG detection kits were purchased from Shanghai Biyuntian Biotechnology Co., Ltd. (Shanghai China). All assays were conducted under standard cell culture conditions (37 °C, 5% CO_2_) with AD-MSCs seeded in 6-well plates. Standards and samples were prepared according to the manufacturer’s instructions, and the absorbance values were measured at 412 nm and 532 nm for GSH and MDA, respectively. The BCA method was used to determine the protein concentrations of the samples.

### 2.6. Evaluation of Mitochondrial Morphology

The treated cells were fixed with an electron microscope fixative, followed by room temperature chemical fixation, dehydration, embedding, and conventional ultra-thin sectioning. Samples were observed using a transmission electron microscope. After dehydration with a series of ethanol solutions and embedding with epoxy resin, ultra-thin sections were prepared and stained with uranyl acetate and lead citrate. Mitochondrial morphology was observed using a transmission electron microscope (JEOL, Tokyo, Japan, Model: JEM-1400Plus).

### 2.7. Western Blotting

We determined the expression levels of GPX4, Nrf2, acyl-CoA synthetase long-chain family member 4 (ACSL4) and HO-1 proteins. AD-MSCs were seeded in 6-well culture plates and treated with corresponding drugs under standard conditions (37 °C, 5% CO_2_); the cells were then scraped from the bottom of the 6-well plates with a cell scraper and collected for protein extraction. The collected cells were lysed on ice for 15 min with RIPA buffer containing protease and phosphatase inhibitors. The lysates were obtained by centrifugation. A BCA kit was used to detect protein levels. All samples were boiled in loading buffer for 5 min to denature the proteins. All samples were subjected to electrophoresis and then transferred to a PVDF membrane. After membrane transfer, the membrane was blocked in 5% fat-free milk powder at room temperature for 1 h and cultured in primary antibodies (Nrf2, GPX4, HO-1, ACSL4, and β-actin) at 4 °C overnight. The dilution ratio of all antibodies was 1:1000, except for GAPDH, which was 1:5000. After rinsing the membrane, the proteins were cultured with the secondary antibody for 1 h (dilution ratio 1:15,000) and finally visualized using chemiluminescence substrate. The antibody information is detailed in Supplementary Table S1. The gel was photographed using a gel-imaging system. The intensity of the protein bands was quantified using Image J 1.53a.

### 2.8. Determination of Migration Ability and Growth Curve of AD-MSCs

Cell migration was detected by wound-healing assay. Passage 4 (P4) cells were seeded into 6-well plates and divided into four groups: DMSO group, RSL3 group, RSL3 + phloretin (25 μM) group, and RSL3 + phloretin (50 μM) group. When cells reached 80–90% confluence, they were treated with corresponding reagents. A uniform scratch was made in the cell monolayer using a sterile pipette tip, and the detached cell debris was washed away with PBS. The scratch was immediately photographed under an inverted microscope equipped with an automated stage (Olympus, Tokyo, Japan, Model: IX73); the same fields of view were marked and re-photographed after 12 h of 37 °C and 5% CO_2_ in a humidified incubator. The scratch area was measured using Image J software, and the migration rate was calculated as follows: Migration rate (%) = (Initial scratch area − Scratch area at the indicated time point)/Initial scratch area × 100%.

For the growth curve assay, P4 AD-MSCs in good condition were seeded into 24-well plates and equally divided into the same four groups. Cells were cultured in medium containing corresponding drugs. Every day, 3 wells from each group were digested with trypsin, and the cell number was counted using a hemocytometer for 5 consecutive days. The growth curve was plotted with time (d) as the abscissa and the average cell count (×10^4^ cells/well) as the ordinate.

### 2.9. Statistical Analysis

Statistical analyses were performed using GraphPad Prism 10.1.2. All data were tested for normality and homogeneity of variance prior to parametric analysis; only qualified data were analyzed by Student’s *t*-test and one-way ANOVA, with significance defined as *, *p* < 0.05; **, *p* < 0.01; ***, *p* < 0.001; ****, *p* < 0.0001. The results are expressed as mean ± standard deviation (mean ± SD). Data were collected from at least three biological replicates and three technical replicates.

## 3. Results

### 3.1. Cell Culture and Identification

**Figure 1:**
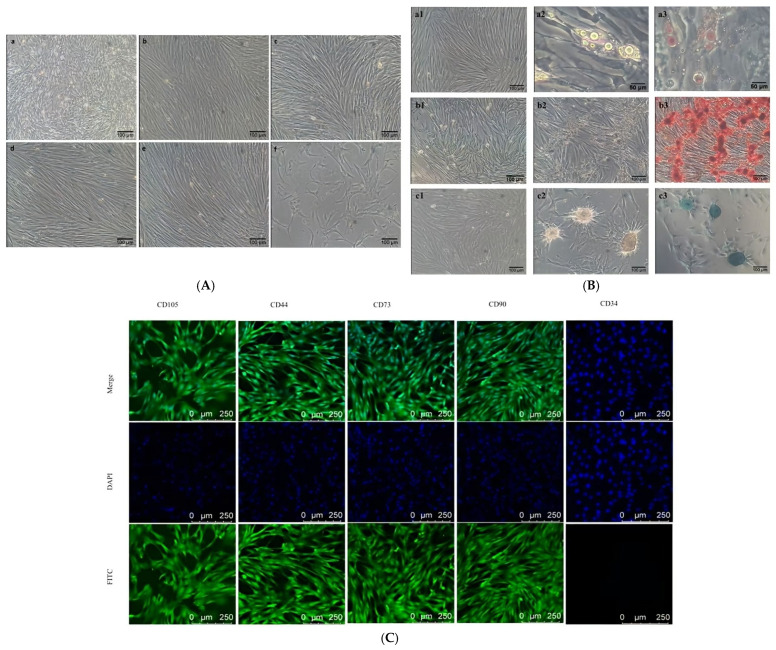
Cell culture and identification of cell types. (**A**) Morphology of cells at different passages: a–f: P0, P3, P4, P5, P6, P11. (**B**) Multilineage differentiation potential assessed by specific staining. Adipogenic induction control group (a1); before adipogenic staining (a2); representative images of Alizarin Oil Red O (orange-red lipid droplets, a3), osteogenic induction control group (b1); before osteogenic staining (b2); Alizarin Red (red indicates mineralized calcium nodules, b3), chondrogenic induction control group (c1); before chondrogenic staining (c2); Alcian Blue (blue represents glycosaminoglycans, c3), staining confirmed osteogenic, chondrogenic, and adipogenic differentiation, respectively. (**C**) Immunofluorescence detection. Columns show fluorescence images of different surface markers. Rows present FITC (green, cell surface markers), DAPI (blue, cell nuclei), and merged images, respectively. The three images in each column were captured from the same field of view under different channels.

The morphology of goat AD-MSCs at representative passages is shown in Figure 1A. Freshly isolated goat AD-MSCs were accompanied by substantial cell debris, appeared round or oval, and were dispersed in the medium. After adherence, the cells became spindle- or fusiform-shaped, were sparsely distributed, and grew in random directions. With increasing cell passages, cell debris gradually diminished and almost disappeared, and the cell population was sufficiently purified by the third passage (P3), with cells displaying a typical spindle shape and arranged in a vortex-like pattern. However, cell proliferation slowed with higher passage numbers, and signs of senescence emerged from passage 11 (P11), such as cytoplasmic vacuolation.

Following induction differentiation, goat AD-MSCs successfully differentiated into adipocytes, osteocytes, and chondrocytes (Figure 1B), indicating that the cells isolated from goat adipose tissue possessed the characteristics of MSCs. Immunofluorescence staining was performed to identify cell surface markers. The MSC surface markers CD105, CD90, CD44, and CD73 were predominantly expressed in the cytoplasm and exhibited green fluorescence, whereas DAPI stained cell nuclei and showed blue fluorescence. Immunofluorescence analysis confirmed that the isolated goat AD-MSCs were positive for CD44, CD73, CD90, and CD105, but negative for the hematopoietic stem cell marker CD34 (Figure 1C).

### 3.2. Effects of RSL3 and Phloretin on Cell Viability

**Figure 2:**
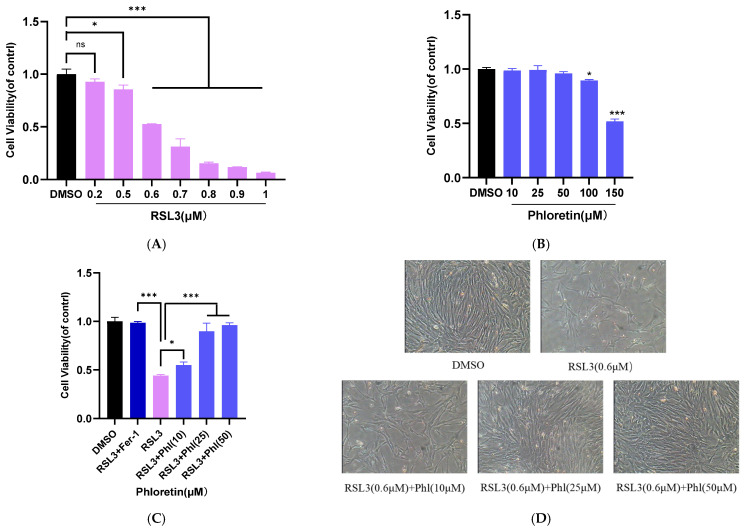
Effects of RSL3 and phloretin on cell viability. (**A**) AD-MSCs were first exposed to different concentrations of RSL3 (0.2, 0.5, 0.6, 0.7, 0.8, 0.9, and 1.0 µM) for 24 h, and cell viability was determined using the CCK-8 assay. (**B**) Effect of different concentrations of phloretin on AD-MSCs viability. (**C**) Cytoprotective effect of phloretin. AD-MSCs were pretreated with varying concentrations of phloretin (10, 25, 50 µM) for 6 h, followed by the addition of RSL3 (0.6 µM), and the treatment was continued for 24 h. Cell viability was measured using the CCK-8 assay. (**D**) Changes in the number of AD-MSCs were observed. All the results are expressed as mean ± SD (*n* = 3); statistical analysis was performed using one-way analysis of variance (ANOVA) followed by Tukey’s post hoc test for multiple comparisons. *, *p* < 0.05; ***, *p* < 0.001; “ns” denotes non-significant (*p* ≥ 0.05).

To determine the effects of RSL3 and phloretin on the viability of goat AD-MSCs, the cells were incubated with varying concentrations of RSL3 and phloretin for 24 h, and cell viability was measured using the CCK-8 assay. The results showed that RSL3 significantly inhibited the proliferation of AD-MSCs in a dose-dependent manner (Figure 2A), whereas phloretin exerted no cytotoxic effects on the cells within an appropriate concentration range (0~50 μM) (Figure 2B). Based on these results, 0.6 μM RSL3 was used in subsequent experiments. To evaluate the cytoprotective effect of phloretin, cells were pretreated with 10, 25, and 50 μM phloretin or Ferrostatin-1 (Fer-1) for 6 h prior to exposure to 0.6 μM RSL3 for another 24 h. The results showed that Fer-1 significantly protected against RSL3-induced cell death, preliminarily confirming the abnormal activation of ferroptosis in RSL3-treated AD-MSCs. Notably, 50 μM phloretin exerted a protective effect similar to that of Fer-1. Compared with the RSL3-treated group, phloretin pretreatment improved the viability of AD-MSCs in a dose-dependent manner (Figure 2C). Accordingly, 25 and 50 μM phloretin were selected for subsequent experiments. The cytoprotective effect of phloretin on RSL3-treated AD-MSCs was further verified by observing changes in cell number (Figure 2D).

### 3.3. Phloretin Inhibits RSL3-Induced Ferroptosis in AD-MSCs

**Figure 3:**
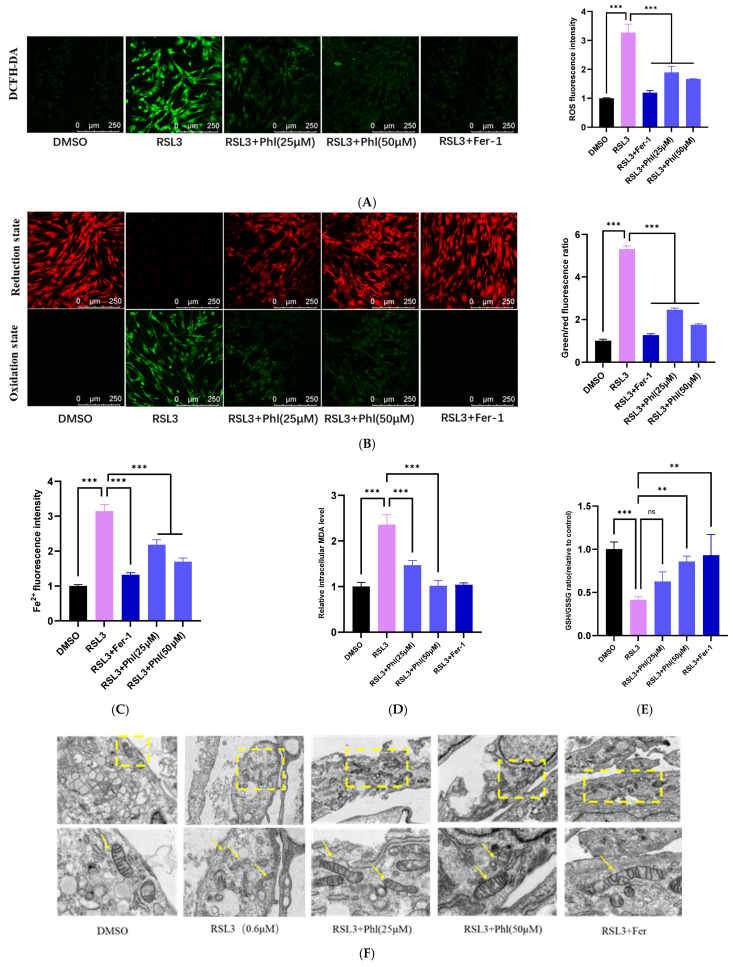
Phloretin inhibits RSL3-induced ferroptosis in AD-MSCs. (**A**) Intracellular ROS levels detected by DCFH-DA probe (fluorescence microplate reader, relative fluorescence intensity). Green fluorescence indicates the intensity of DCF, representing intracellular ROS levels. (**B**) Lipid peroxidation levels measured by C11-BODIPY fluorescent probe (fluorescence microplate reader, ratio of green/red relative fluorescence intensity), red fluorescence represents non-oxidized lipids, while green fluorescence represents oxidized lipids. A higher green/red fluorescence ratio indicates more severe lipid peroxidation. (**C**) Fe^2+^ was detected using the FerroOrange probe; (**D**) cellular MDA levels were determined; (**E**) cellular GSH/GSSG levels were determined; (**F**) TEM was performed to evaluate the microscopic changes in mitochondria. The structure in the yellow box is the mitochondrion and the yellow arrows denote mitochondria (2 μM and 500 nM). All the results are expressed as mean ± SD; statistical analysis was performed using one-way analysis of variance (ANOVA) followed by Tukey’s post hoc test. **, *p* < 0.01; ***, *p* < 0.001, “ns” denotes non-significant (*p* ≥ 0.05).

To further evaluate the exact effect of phloretin on RSL3-induced ferroptosis in AD-MSCs, we conducted a series of assays. Intracellular ROS levels were detected using the DCFH-DA probe, lipid peroxidation was assessed with the BODIPY-C11 probe, and Fe^2+^ levels were measured using the FerroOrange probe. The data indicated that compared with the control group, the intracellular Fe^2+^, ROS, and lipid peroxidation product levels in the RSL3 treatment group were markedly increased, whereas the expression of these products in the phloretin pretreatment group and Fer-1 pretreatment group were significantly decreased (Figure 3A–C). Furthermore, the detection found that phloretin treatment significantly inhibited the accumulation of intracellular MDA induced by RSL3 (Figure 3D). Moreover, the decrease in the GSH/GSSG ratio caused by RSL3 stimulation was rescued by phloretin (Figure 3E). To further verify the ferroptosis-promoting effect of phloretin on AD-MSCs, mitochondrial morphological alterations were examined by TEM. The images revealed that RSL3 treatment led to mitochondrial shrinkage, damaged or even disappeared mitochondrial cristae, and ruptured outer membranes in AD-MSCs. However, phloretin significantly mitigated these RSL3-induced mitochondrial morphological changes in AD-MSCs (Figure 3F), and these effects were comparable to those of pretreatment with Fer-1.

### 3.4. Phloretin Regulated Nrf2/HO-1/GPX4 Signaling Pathway to Improve Ferroptosis

**Figure 4:**
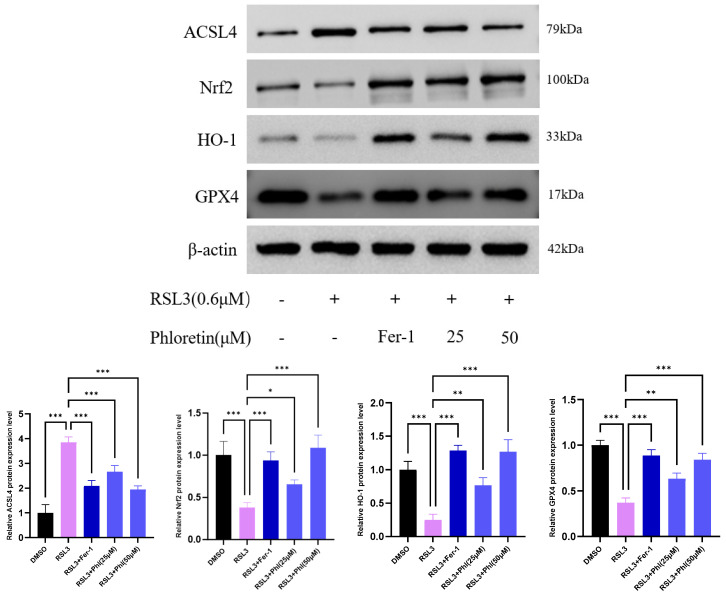
Phloretin regulates the Nrf2/HO-1/GPX4 signaling pathway to improve ferroptosis. ADMSCs were pretreated with phloretin (25 and 50 µM) for 24 h and then treated with 0.6 µM RSL3 for 24 h. The expression levels of ACSL4, Nrf2, HO-1, and GPX4 were measured using Western blotting assay. β-actin was used as an internal control for total protein. All results are expressed as mean ± SD; statistical analysis was performed using one-way analysis of variance (ANOVA) followed by Tukey’s post hoc test. *, *p* < 0.05; **, *p* < 0.01; ***, *p* < 0.001.

Our results showed that phloretin treatment significantly restored the reduced GSH/GSSG ratio induced by RSL3. To investigate the mechanism by which phloretin inhibits ferroptosis in goat AD-MSCs, Western blotting was employed to detect the expression of ferroptosis-related proteins. The results showed that RSL3 treatment significantly decreased the intracellular protein expression of Nrf2, HO-1, and GPX4 and increased the expression of the ferroptosis protein marker ACSL4. Compared with the RSL3 group, the protein expression levels of Nrf2, HO-1, and GPX4 in the two-dose phloretin treatment groups and Fer-1 positive control group were significantly increased, whereas the expression of ACSL4 was significantly decreased (Figure 4). These results suggest that phloretin may alleviate ferroptosis in goat AD-MSCs by regulating the Nrf2/HO-1/GPX4 signaling pathway.

### 3.5. Phloretin Improves the Functions of AD-MSCs Under Ferroptosis Induction

**Figure 5:**
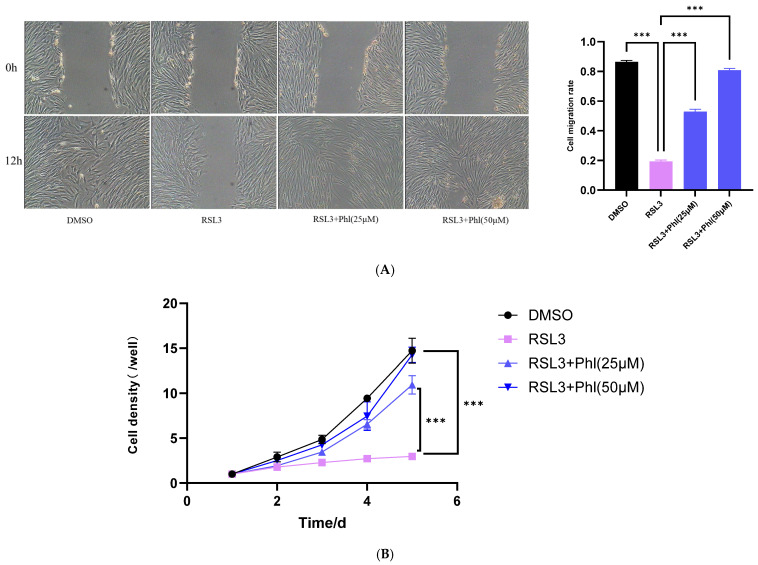
Effects of phloretin on the growth curves and migration ability of ferroptosis-induced goat AD-MSCs. (**A**) The migration and repair capacity and migration rate of cells in each group detected by wound-healing assay (bar = 100); (**B**) growth curves showing the growth status of cells in each group after different treatments. Cell number was counted in triplicate wells per group per time point. Data were collected from three biological replicates. Statistical analysis was performed using two-way ANOVA. ***, *p* < 0.001.

In the above experiments, we have demonstrated that phloretin can significantly ameliorate the decrease in cell viability induced by ferroptosis. Next, we investigated the protective effect of phloretin on cellular functions impaired by ferroptosis. In the wound-healing assay, ferroptosis induction markedly suppressed cell migration and delayed scratch closure. Notably, phloretin pretreatment significantly enhanced the migration ability of goat AD-MSCs in a dose-dependent manner and increased the scratch closure rate compared with the model group (*p* < 0.01) (Figure 5A). The growth curve assay showed that ferroptosis induction significantly reduced the proliferation rate and cell number of goat AD-MSCs compared with the control group. In contrast, pretreatment with phloretin markedly alleviated the ferroptosis-induced inhibition of cell proliferation and increased cell viability, and the growth curve was significantly restored (Figure 5B). These results demonstrate that phloretin effectively restores the proliferation and migration functions of goat AD-MSCs by inhibiting ferroptosis, thereby exerting a protective effect on these cells.

## 4. Discussion

Biotechnological strategies combining AD-MSCs with natural bioactive small molecules offer favorable economic efficiency and operability for enhancing livestock and poultry production. Unlike gene editing or transgenesis—technologies with high technical barriers—phloretin can serve as a preconditioning agent for AD-MSCs prior to in vitro transplantation. This approach features simple operation, high biosafety, and minimal reliance on specialized equipment, thereby avoiding significant increases in costs associated with molecular or stem cell-assisted breeding. By focusing on cellular protection, this preconditioning strategy bypasses the need for multi-generational selection and is applicable to the optimization of stem cell in vitro expansion and transplantation systems across various breeding platforms, and thus exhibits strong potential for promotion and application. For local characteristic breeds such as Leizhou goats, although they receive less attention in commercial breeding systems than specialized high-yield breeds, inhibiting ferroptosis and maintaining the functional homeostasis of AD-MSCs can effectively enhance the activity of stem cells after in vitro manipulation and their in vivo engraftment efficiency, thereby providing technical support for the genetic improvement of local breeds.

The survival and functional maintenance of MSCs are critically important for animal growth, development, tissue repair, and regeneration. In vivo studies conducted by a research team have confirmed that MSCs in the mammary gland microenvironment not only protect mammary epithelial cells from oxidative stress damage via paracrine effects but also effectively promote milk fat synthesis [23]. In addition, in vivo studies in the reproductive field have also found that AD-MSCs can improve oocyte quality and support endometrial repair and regeneration [7]. Furthermore, AD-MSCs can facilitate ovarian and endometrial repair, improve oocyte quality, promote embryonic development and restore reproductive function through multiple pathways, including homing to injured sites, secreting bioactive factors, and regulating local immune responses [24]. Ferroptosis, a distinct form of programmed cell death, has been identified in transplantation-related in vitro studies as a crucial factor restricting the efficiency of MSC transplantation and their functional performance in vivo [25]. Ferroptosis-induced MSC damage can markedly reduce the efficacy of MSCs in breeding and production applications, thereby affecting the overall efficacy of molecular breeding and stem cell-assisted breeding in ruminants.

Our study is the first to demonstrate that phloretin exerts a significant inhibitory effect on ferroptosis in goat AD-MSCs, and it exerts its cytoprotective roles through three key pathways: the Nrf2/HO-1/GPX4 antioxidant axis, iron homeostasis regulation, and lipid peroxidation scavenging. These findings provide novel molecular targets and a natural regulatory strategy for molecular breeding and stem cell-assisted breeding focused on optimizing the AD-MSCs in vitro manipulation system with cellular survival and oxidative homeostasis as the core targets. They also possess potential translational application value for stem cell-assisted breeding research of other ruminant species.

Ferroptosis is a form of programmed cell death caused by the accumulation of lipid peroxidation products which is mainly regulated by the Nrf2 signaling pathway. The main manifestations of ferroptosis include inactivation of GPX4 in cells, increased levels of Fe^2+^ and lipid peroxidation, accumulation of ROS, and excessive consumption of GSH [24,26,27]. As a key selenoprotein, GPX4 uses GSH to catalyze the reduction in harmful lipid peroxides to inactive lipid alcohols, thereby preventing the propagation of lipid peroxidation chain reactions [28]. As a direct inhibitor of GPX4, RSL3 can perfectly simulate this process, leading to a sharp increase in intracellular lipid peroxidation levels, ultimately triggering cell death [29]. In vitro cell-based studies have found that phloretin can inhibit the production and accumulation of lipid peroxides and MDA in RSL3-induced 293T cells and suppress the expression of the GPX4 signaling pathway induced by RSL3, thereby inhibiting ferroptosis and improving cell viability [30]. To explore the protective effect of phloretin on MSC ferroptosis and its potential molecular mechanism, goat AD-MSCs were treated with different concentrations of RSL3 to establish a ferroptosis model. The results showed that RSL3 effectively inhibited the proliferation of AD-MSCs, and phloretin significantly alleviated this inhibition. In addition, we found that the Fer-1 significantly alleviated the inhibitory effect of RSL3 on AD-MSC viability. This result initially indicated that RSL3 treatment successfully established a ferroptosis model in goat AD-MSCs, and phloretin has a protective effect against this type of cell death.

Further detection results showed that RSL3 treatment significantly increased intracellular Fe^2+^, ROS, lipid peroxidation, and MDA levels in goat AD-MSCs, while it decreased GSH content, consistent with typical ferroptotic characteristics. Moreover, the results clearly showed that phloretin could effectively alleviate these changes in cells caused by RSL3 in a dose-dependent manner, which is comparable to the protective effect of Fer-1, a classic ferroptosis inhibitor [25]. This study is the first to confirm in goat AD-MSCs that phloretin can effectively alleviate RSL3-induced ferroptosis. The reduced cell viability, elevated lipid peroxidation, and downregulated GPX4 expression induced by RSL3 were significantly reversed by phloretin, suggesting that its protective effect is closely related to the direct inhibition of the ferroptosis pathway. This provides core experimental evidence supporting phloretin as a promising preconditioning agent for AD-MSCs.

Multiple in vitro studies have shown that phloretin has strong antioxidant and free radical-scavenging functions [24]. GPX4 is a core regulatory factor in the ferroptosis pathway, responsible for reducing toxic lipid peroxides to non-toxic lipid alcohols, thereby maintaining cell membrane integrity [31,32]. RSL3 inactivates GPX4 by covalently binding to its active site, directly inducing ferroptosis [33], and this conclusion from in vitro studies is also consistent with the results of this study. Western blot results in this study showed that phloretin significantly reverses the RSL3-induced downregulation of GPX4 protein expression. Based on these observations, the protective effect of phloretin against RSL3-triggered injury is mainly mediated through its direct antioxidant activity. By neutralizing lipid peroxyl radicals and hydroxyl radicals, phloretin interrupts the lipid peroxidation chain reaction, alleviates oxidative depletion and damage to GPX4, and thereby maintains the structural and functional stability of the cell membrane. This action pathway has also been supported by in vitro studies and serves as an important mechanism for phloretin as a preconditioning agent for AD-MSCs [16].

In vitro studies have confirmed that natural products can resist ferroptosis by activating the endogenous antioxidant defense system [34]. This study found that phloretin significantly upregulated Nrf2 protein expression. Combined with the existing in vitro evidence that phloretin activates the Nrf2 pathway in various cell types, these results suggest that phloretin may exert its anti-ferroptotic effect through the Nrf2/GPX4 axis [35]. Nrf2 is a key transcription factor in the cellular antioxidant response that regulates the expression of multiple proteins involved in GSH synthesis and antioxidant defense, including HO-1, solute carrier family 7 member 11 (SLC7A11), and GPX4. It also plays an important role in maintaining the stability and function of GPX4 [36,37]. This regulatory relationship has also been clarified by previous studies, and this pathway is expected to become an important research direction for regulation ferroptosis in AD-MSCs, providing new targets for optimizing stem cell in vitro preconditioning protocols.

Meanwhile, this study confirmed that after phloretin treatment, the expression level of the HO-1 protein in goat AD-MSCs was significantly increased. This indicates that the protective effect of phloretin depends not only on direct antioxidant activity but also the enhancement of endogenous cellular antioxidant capacity and overall stress resistance via activation of the Nrf2/HO-1 pathway. This regulatory mechanism has also been verified through in vitro studies [38]. In vitro cell model studies have confirmed that Nrf2 pathway activation can effectively inhibit ferroptosis [39]. Based on the above results, phloretin effectively antagonizes RSL3-induced ferroptosis in AD-MSCs through a dual mechanism: direct antioxidant activity and activation of the Nrf2/HO-1/GPX4 pathway. This provides complete molecular mechanistic support for its application as a preconditioning agent for AD-MSCs prior to in vitro transplantation [39].

In addition, the iron ion chelating ability of phloretin may also contribute to its protective effect. Ferroptosis relies on the Fenton reaction mediated by intracellular Fe^2+^ to generate a large number of free radicals [40], while the polyphenolic hydroxyl groups in the phloretin structure endow it with the potential to chelate metal ions, a characteristic that has also been clarified by in vitro experiments [41]. Our results showed that after RSL3 induction, the activity of AD-MSCs decreased, accompanied by increased numbers of FerroOrange-positive cells and enhanced fluorescence intensity, indicating elevated intracellular iron levels. However, phloretin pretreatment significantly reversed this change. This suggested that phloretin may inhibit the initiation of lipid peroxidation by chelating Fe^2+^ in the labile iron pool and reducing hydroxyl radical generation, further refining its mechanism of action as an AD-MSC preconditioning agent.

Ferroptosis can significantly impair cell survival and biological function. The proliferation and migration abilities of MSCs are critical for their in vivo homing and tissue repair, and present core cellular characteristics determine the efficiency of stem cell-assisted breeding. This study found that ferroptosis could significantly inhibit the proliferative and migratory capacities of goat AD-MSCs, confirming that ferroptosis is an important cause of the decreased stem cell function after in vitro manipulation; phloretin preconditioning could obviously improve the proliferation inhibition and migratory capacity decline caused by ferroptosis, indicating that it can maintain the basic biological functions of AD-MSCs by inhibiting ferroptosis, which lays a foundation for improving the homing and repair efficiency of AD-MSCs after in vivo transplantation and also provides key experimental data for optimizing the stem cell in vitro preconditioning system and enhancing the efficiency of molecular breeding and stem cell-assisted breeding.

Unlike single antioxidants, phloretin possesses multiple biological activities, including iron chelation, antioxidant activity, anti-inflammation, and mitochondrial protection, making it more suitable for oxidative stress-prone processes such as AD-MSC in vitro expansion and pre-transplantation pretreatment [9]. Compared with gene editing, natural small molecule pretreatment has the advantages of safety, low cost, and easy industrialization, and can serve as a core auxiliary strategy for AD-MSC in vitro expansion, somatic cell nuclear transfer, and gene-editing breeding. By improving the survival ability and functional integrity of in vitro AD-MSCs, it can increase the embryonic development rate and cell transplantation survival rate, shorten the breeding cycle, and enhance the overall efficiency of molecular breeding and stem cell-assisted breeding. At present, improving the production performance and stress resistance of ruminants is the core goal of genetic breeding. Traditional breeding has a long cycle and low efficiency, while precision breeding combining stem cell-assisted breeding with molecular markers has become a research hotspot. This study confirmed at the cellular level that phloretin can maintain AD-MSC function by inhibiting ferroptosis, suggesting that ferroptosis-related genes such as Nrf2, GPX4, and SLC7A11 can serve as important candidate genes for stress resistance traits in ruminants. In the future, these genes can be used for the molecular regulation of the AD-MSCs in vitro manipulation system to accelerate the breeding of new high-yield and stress-resistant ruminant lines, providing new molecular targets for molecular breeding and stem cell-assisted breeding of ruminants.

This study clarified the protective effect of phloretin against ferroptosis in goat AD-MSCs and provided a novel preconditioning strategy for improving stem cell survival capacity under stress conditions. In addition, during stem cell therapy, transplanted MSCs are exposed to high oxidative stress environments, such as ischemia, hypoxia, and inflammation, which easily induce ferroptosis, leading to massive cell death and unsatisfactory therapeutic effects [41]. Based on the results of this study, phloretin is expected to serve as a high-efficiency preconditioning agent for AD-MSCs prior to transplantation, improving the in vivo transplantation survival rate and functional efficiency of MSCs and expanding their application in the fields of reproductive health, tissue repair, and genetic breeding of ruminants.

Our study has several limitations: the protective effect of phloretin on goat AD-MSCs and the related mechanisms were only verified at the in vitro cell level, and there is a lack of in vivo animal experiments to further verify the in vivo engraftment efficiency and functional exertion of AD-MSCs after phloretin preconditioning; the direct regulatory effect of phloretin-preconditioned AD-MSCs on economic traits related to ruminant breeding has not been thoroughly analyzed; and the crosstalk between signaling pathways and long-term effects need to be further explored. In the future, in vivo transplantation experiments can be combined to verify the in vivo functions of phloretin-preconditioned AD-MSCs; gene knockdown and overexpression technologies can be used to further analyze the regulatory details of the Nrf2/HO-1/GPX4 pathway; meanwhile, the in vitro preconditioning process of phloretin can be optimized to provide technical support for its practical application in molecular breeding and stem cell-assisted breeding of ruminants.

In summary, this study elucidates the protective effect of phloretin against ferroptosis in goat AD-MSCs and confirms that it exerts an anti-ferroptotic role by regulating the Nrf2/HO-1/GPX4 signaling pathway. Meanwhile, phloretin effectively alleviates the impaired proliferation and migration of AD-MSCs induced by ferroptosis. The findings provide a theoretical basis and experimental support for improving the in vitro culture efficiency and transplantation survival rate of AD-MSCs in goats and other ruminants, and also lay a foundation for the development and application of phloretin as a preconditioning agent for AD-MSCs prior to in vitro transplantation in molecular breeding and stem cell-assisted breeding of ruminants, providing new ideas and technical means for the precision breeding of ruminants. Further in vivo experiments and translational studies are warranted to validate the efficacy and safety of phloretin.

## 5. Conclusions

In conclusion, we established an RSL3-induced ferroptosis model in goat AD-MSCs in vitro and demonstrated that phloretin reverses ferroptotic alterations and alleviates the inhibitory effects of RSL3 on cell proliferation and migration in a dose-dependent manner. Phloretin exerts anti-ferroptotic effects via direct antioxidant activity, activation of the Nrf2/HO-1/GPX4 signaling pathway, and Fe^2+^ chelation, with Nrf2 and GPX4 as key targets. These findings provide in vitro experimental evidence and theoretical support for developing phloretin as a green pretreatment agent for AD-MSC transplantation and offer potential molecular targets and novel strategies for ruminant precision breeding.

## Data Availability

Data are contained within the article.

## Supplementary Materials

The following supporting information can be downloaded at: https://www.mdpi.com/article/10.3390/ani16091286/s1, Table S1: Antibodies used for Western blot analysis.
