# Supplementary material for: Phloretin Protects Goat Adipose-Derived Mesenchymal Stem Cells Against Ferroptosis by Regulating the Nrf2/HO-1/GPX4 Signaling Pathway
Source: Animals (Basel). 2026 Apr 22;16(9):1286. doi: 10.3390/ani16091286 (PMC13162607; doi:10.3390/ani16091286)
Supplement: Supplementary file 1 [file animals-16-01286-s001.zip › animals-4183511-supplementary.pdf]

Supplementary Table S1. Antibodies used for Western blot analysis.

**Antibody name**	**Manufacturer**	**Catalog No.**	**MW（kDa）**	**Species**	**Dilution ratio**
Nrf2	HUABIO	HA723302	100	rab	1:2000
HO-1	Proteintech	10701-1-AP	33	rab	1:2000
SLC7A11	Abways	CY7046	55	rab	1:50,000
ACSL4	Abways	DY1198	79	rab	1:2000
GPX4	Abways	CY6959	17	rab	1:2000
β-actin	Servicebio	GB11001	42	rab	1: 15,000
Goat anti-Rabbit IgG(H+L)	Sera care	5220-0336			1：15,000
